# Dysregulation of PI3K-Akt-mTOR pathway in brain of streptozotocin-induced type 2 diabetes mellitus in Wistar rats

**DOI:** 10.1186/s12944-018-0809-2

**Published:** 2018-07-24

**Authors:** Siresha Bathina, Undurti N. Das

**Affiliations:** 1 BioScience Research Centre, Department of Medicine, Gayatri Vidya Parishad Hospital, GVP College of Engineering Campus, Visakhapatnam, 530048 India; 2 UND Life Sciences, 2221, NW 5th St, Battle Ground, WA 98604 USA

**Keywords:** Streptozotocin, PI3K, Insulin signaling, β-Cell survival

## Abstract

**Background:**

Proteins of the insulin signaling pathway are needed for cell proliferation and development and glucose homeostasis. It is not known whether insulin signalling markers (Foxo1, Gsk3β) can be correlated with the expression on PI3K-Akt-mTOR pathway, which are needed for cell survival and maintenance of glucose homeostasis. In the present study, we studied the expression of Foxo1, Gsk3β and PI3K-Akt-mTOR in the brain of streptozotocin-induced type 2 diabetes mellitus Wistar rats.

**Methods:**

The study was performed both in vitro (RIN5F cells) and in vivo (male Wistar rats). Gene expression of Nf-kB, IkB, Bax, Bcl-2 and Pdx1 gene was studied invitro by qRT-PCR in RIN5F cells. In STZ (65 mg/kg i.p.)-induced type 2 DM Wistar rats, blood glucose and insulin levels, iNOS, Foxo1, NF-κB, pGsk3β and PPAR-γ1 levels along with PI3k-Akt-mTOR were measured in brain tissue.

**Results:**

RIN5F cells treated with STZ showed increase in the expression of NF-kB and Bax and decrease in IkB, Bcl-2 and PDX1. Brain tissue of STZ-induced type 2 DM animals showed a significant reduction in secondary messengers of insulin signalling *(*Foxo1) (*P < 0.001)* and Gsk3β (*P < 0.01)* and a significant alteration in the expression of phosphorylated-Akt (*P < 0.001)* mTOR (*P < 0.01)* and PI3K.

**Conclusion:**

These results suggest that STZ induces pancreatic β cell apoptosis by enhancing inflammation. Significant alterations in the expression brain insulin signaling and cell survival pathways seen in brain of STZ-treated animals implies that alterations neuronal apoptosis may have a role in altered glucose homeostasis seen in type 2 DM that may also explain the increased incidence of cognitive dysfunction seen in them.

## Highlights of the study

- Streptozotocin (STZ)- induced cytotoxicity to RIN5F (rat pancreatic β) cells in vitro is associated with a significant (*P* < 0.001) increase in the expression of Nf-kB, Bax and decrease in IkB-β, Bcl-2 and pancreatic homeobox gene1(Pdx1)*.*
- Protein expression of signalling markers of cell survival (phosphorylated PI3K, Akt and mTOR) and secondary messengers of insulin signaling (pGsk-3β and Foxo1) were also significantly reduced (*P* < 0.001) in the brain of STZ-treated rats.
- Thus, altered glucose homeostasis seen in in STZ-induced type 2 DM could be attributed to alterations in insulin signalling pathway proteins Akt and mTOR in the brain that may also explain neurodegeneration that is common in diabetes mellitus.

## Background

Streptozotocin (STZ)-induced type 2 DM (T2DM) model can be used to study not only the pathobiology of diabetes but also its secondary complications such as diabetic retinopathy [1], neuropathy and vasculopathy [2]. Long-standing diabetes can also cause impaired cognitive function, associated with anxiety [3], depression [4] and memory impairment [2] because of involvement of the central nervous system, a condition referred to as diabetic encephalopathy [5, 6]. In a previous study [7], we observed that neurodegeneration occurs in the CA1, 2/3 (Cornu ammonis) and DG [dentate gyrus) areas of hippocampus and PVN (paraventricular nucleus) of hypothalamus in STZ-induced diabetic animals. Studies have shown that apoptosis is a potential mechanism underlying neuronal death in diabetes mellitus [8]. The PI3K/Akt/mTOR pathway is an intracellular signaling pathway that plays a critical role in the regulation of cell cycle including cellular quiescence, cell proliferation, cancer, and longevity. PI3K activation phosphorylates and activates Akt, localizing it in the plasma membrane [9]. Akt downstream events include: activating CREB [10], inhibiting p27localizing Foxo1 in the cytoplasm, activating PtdIns-3 ps (phosphatidyl inositol 3-phosphate) and activating mTOR, which can affect transcription of p70 or 4EBP1 [11, 12]. Of the several factors that are known to enhance the PI3K/Akt pathway, insulin is an important inducer [11]. This pathway is necessary to promote growth and proliferation over differentiation of adult stem cells neural stem cells [13]. In addition, this pathway is an essential component in neural long term potentiation [14, 15]. Hence, interference with PI3K/Akt/mTOR pathway may lead to apoptosis of neurons and cause memory impairment.

The phosphatidylinositol 3-kinase (PI3K) signalling pathway promotes cell survival and has been reported to participate in apoptosis in the central nervous system. Akt, a serine/threonine protein kinase that is also known as protein kinase B (PKB), is the primary protein effector downstream of the PI3K signalling pathway. Akt has an important role in glucose metabolism by regulating the biological function of insulin by translocation of glucose transporter type 4 (GLUT4) to the plasma membrane, thereby mediating glucose uptake and phosphorylates to inhibit the activity of glycogen synthase kinase 3, which increases the activity of glycogen synthase and promotes glycogen synthesis [15–17]. Furthermore, Akt being the most important apoptosis-inhibiting protein, experimental studies have shown that Akt signalling pathway is involved in the pathophysiological processes of diabetes mellitus and its complications [18–21]. Hippocampus has an important role in cognitive function but the mechanism by which diabetes mellitus induces neurodegeneration has remained elusive.

Deceased intake or deficiency of n-3 PUFAs has been linked to higher incidence of T2DM (21]. Several studies suggested that supplementation of n-3 PUFAs can improve insulin sensitivity [22–24] and reduced macrophage accumulation in adipose tissue [25] DHA administration promotes lateral segregation and alter the composition of cholesterol rich microdomains known as lipid rafts which serve as membrane platforms for multiple signaling events [26]. Such DHA-dependent actions have been associated with altered protein rearrangement and co-localization in these important membrane compartments leading to altered downstream events [26–28] including activating PI3k-Akt-mTOR signaling in brain. But, it is not clear how exactly n-3 PUFAs and other fatty acids are able to prevent obesity-linked inflammation and insulin resistance and what could be mechanisms involved in inducing these beneficial actions.

Cognitive dysfunction and diabetes are parallel phenomenon arising from coincidental roots linked to various pathological changes such as alterations in anti-oxidants SOD, CAT, GST and enhancement of inflammatory markers TNF-α, IL-6, etc. In the brain, insulin receptor density is highest in the olfactory bulb, hypothalamus, hippocampus, cerebral cortex, striatum and cerebellum [29, 30] which mediates translocation of glucose transporter (GLUT-4) and regulate memory formation and other cognitive functions by activation of phosphorylated Gsk-3β, cAMP/CREB involved in cell survival.

In the present study, we sought to delineate the molecular alterations that could occur in the brain of STZ-induced type 2 DM animals by analyzing expressions of key proteins of the insulin pathway especially those that are involved in cellular proliferation, such as PI3K / Akt / mTOR. It is known that PI3K activation phosphorylates and activates AKT, localizing it in the plasma membrane [31]. AKT, in turn, activates PtdIns-3 ps, and mTOR [32]. Insulin is one of the important factors that enhances the PI3K/AKT pathway. The pathway is necessary to promote growth and proliferation of neural stem cells [33]. Hence, we evaluated in the present study the effect of STZ on PI3K/AKT/mTOR pathway in the brain.

These results revealed significant reduction in phosphorylated form of Akt and TOR along with downstream signaling molecule Gsk-3β. The serine/threonine kinase Akt, a downstream target of phosphatidylinositol-3 kinase (PI3K], which is transiently phosphorylated at Ser473 was downregulated in STZ-treated animals. These results suggest that STZ induces neuronal toxicity via dysregulation of PI3K-Akt signaling pathway and pGSK3β inhibition.

## Methods

### Chemicals

All chemicals (including STZ) were purchased from Sigma Aldrich chemical company, USA. PCR reagents were obtained from Genei, (Bangalore, India). PCR primers were purchased from Bioserve, (Hyderabad, India).

### Cell culture

RIN5F cells, insulin secreting rat pancreatic β cell line, were obtained from National Center for Cell Science (Pune, India). RIN5F cells were grown in RPMI1640 medium supplemented with bicarbonate (1.5 g/L), Glucose (4.5 g/L) 10% FBS, Penicillin (100 U/ml), Streptomycin (100 μg/ml), Amphotericin B (1.25 μg/ml), HEPES (10 Mm) and L-Glutamine (2 mM) at 37 °C in humidified air with 5% CO_2_. After 48 h of incubation time, cells reached confluent stage at which time they were harvested for further studies [34, 35].

### Cell viability assay

#### Dose and time optimization studies with STZ on RIN5F cells

RIN5F cells were seeded at a density of 5 X 10^4^ cells /100 μl of culture media in 96-well plates. STZ was dissolved in 100 mM citrate buffer (pH 4.5). After 44 h of attachment period, cells were treated with different doses of STZ (1 mM–30 mM) and incubated from 12 to 48 h. At the end of incubation, Cell viability assay was performed by MTT (3, 4, 5-dimethylthiazol-2-yl)-2–5-diphenyl tetrazolium bromide) assay [7, 34–36].

#### Effect of BDNF on STZ-induced cytotoxicity to RIN5F

This study was performed with 5 X 10^4^ cells /100 μl of culture media in 96-well plates. The RIN5F cells were treated with optimal doses of BDNF and STZ (20 mM) simultaneously to study the effect of BDNF on the cytotoxic action of STZ against RIN5F cells and incubated for optimized period. At the end of the incubation period, viability of cells was assessed by MTT assay.

### Estimation of nitric oxide, lipid peroxides and antioxidant enzymes in vitro

For this study, RIN5F cells were seeded at a density of 1 X 10^6^ cells/1 ml/well in 6 well culture plates. After 48 h of attachment period, cells were treated with STZ (20 mM) for 24 h. At the end of the treatment, cells were harvested and used for analysis of their anti-oxidant content. At the end of the treatment period, the spent media was collected, and cells were washed with PBS (pH 7.4). The washed cells were lysed with lysis buffer (20 mM Tris, 100 mM NaCl, 1 mM EDTA and 0.5% of 10% Triton-X) and the lysates were used for the measurement of various antioxidant enzymes. In this study, the following anti-oxidant enzymes were measured: catalase, superoxide dismutase, glutathione-S-transferase and glutathione peroxidase. Lipid peroxides and nitric oxide were measured both in the cell culture supernatants and RIN5F cells as described previously [7, 34–36].

### Effect of STZ on the levels of lipoxinA4 in vitro

RIN5F cells were cultured in 24 well culture plates at a concentration of 0.5 × 10^6^/well in 500 μl of RPMI1640 and treated with optimal dose of STZ (20 mM) and BDNF (100 ng/ml) for 24 h. At the end of incubation period, the supernatant was collected and their content of BDNF was measured by Elisa(EA45) according to manufacturer instructions.

### Gene expression studies

#### Isolation of RNA and cDNA synthesis

RNA was isolated by homogenizing pancreatic and brain tissues using Trizol reagent; cDNAs were then synthesized by reverse transcription from 1 μg of total RNA using SuperScript First Strand Synthesis for qRT-PCR (Invitrogen). Both the experiments (RNA isolation and qRT-PCR) were done per the manufacturer’s instructions.

#### Semi-quantitative PCR for gene expression studies

The cDNAs used to study the expression of all the six genes: *P65 Nf-kB/IkB-β/Bcl2/Bax/Pdx* and *β-Actin* obtained from Bioserve, India. For *Nf-κB* (forward, *5’-CCTAGCTTTCTCTGAACTGCAAA-3′*; reverse *5′- GGGTCAGAGGCCAATAGAGA-3′*) the product size of 71 bp and for *IKB-β* (forward, *5’-TGGCTCATCGTAGGGAGTTT-3′*; reverse *5’-CTCGTCCTCGACTGAGAAGC-3′*) the product size of 69 bp was used. For *Bcl2* gene (forward, *5’-CACCCCTGGCATCTTCTCCTT-3′*; reverse *5’-AGCGTCTTCAGAGACAGCCAG-3′*) the product size of 110 bp and for *Bax* gene (forward *5’-CACCAGCTCTGAACAGATCATGA-3′*;reverse *5’-TCAGCCCATCTTCTTCCAGATGT-3′*) the product size of 105 bp were used. PCR was performed as follows: 94 °C for 2 min initial denaturation, 94 **°**C for 30 s denaturation, 64 °C and 60 °C for 30s annealing for P65 *Nf-κB* and *IKB*-*β* respectively; 94 °C for 2 min initial denaturation, 95 **°**C for 30s denaturation, 59 °C for 30s annealing for *Bcl2* and *Bax*. Later 72 °C for 30s extension and 72 **°**C for 5 min final extension, and overall 35 cycles (34 cycles for *Bcl2/Bax*) were performed. For *Pdx* gene expression studies, (forward, *5’-GTAGTAGCGGGACAACGAGC-3′*; reverse *5′- CAGTTGGGAGCCTGATTCTC-3′*) the product size of 528 bp and for *β-actin* (forward*,5’-CGTGGGCCGCCCTAGGCACCA-3′*; reverse *5’-TTGGCCTTAGGGTTCAGGGGG-3′)* the product size of 617 bp were used. PCR was performed as follows: 94 °C for 2 min initial denaturation, 94 **°**C for 30s denaturation, 65 °C and 52.5 °C for 30s annealing for *Pdx* and *β-actin* respectively and 72 °C for 30s extension with 72 **°**C for 5 min final extension, and overall 35 cycles were performed. PCR products were run on 1.5% (*w*/*v*) agarose gel in 1× TAE buffer by electrophoresis at 100 V. PCRs were run on an Eppendorf 5331 Master cycler gradient (96well). Quantification of genes was done by Major Science image analysis software. Thermal cycler in which individual amplification reactions are set in a designed program. All experiments were done in triplicate, and statistical analysis was performed based on the ratio of gene of interest transcripts and the amounts of β-actin and calculating as percentage comparing with respective control.

### In vivo experiment

Male Wister rats, 3 to 4wk old, purchased from National Institute of Nutrition, (Hyderabad, India) were used for this study. The animals were housed at 25^0^ C room temperature with 12-h dark and 12-h light cycle. Animals weighing around 180 g were segregated into two groups of 6 animals: controls received PBS and citrate buffer; diabetic group received STZ dissolved in citrate buffer. This study was approved by Institutional Animal Ethical Committee.

#### Induction of type 2 diabetes mellitus

Type 2 DM was induced by treatment with nicotinamide (NAD) and STZ. Freshly prepared 175 mg/kg body weight nicotinamide in PBS was administered intraperitoneally. After 15mins freshly prepared 65 mg/kg body weight STZ in 50 mM citrated buffer pH 4.5 was injected intraperitoneally as described previously [34, 35].

#### Estimation of fasting blood glucose and insulin and body weight measurement

**Fig. 1:**
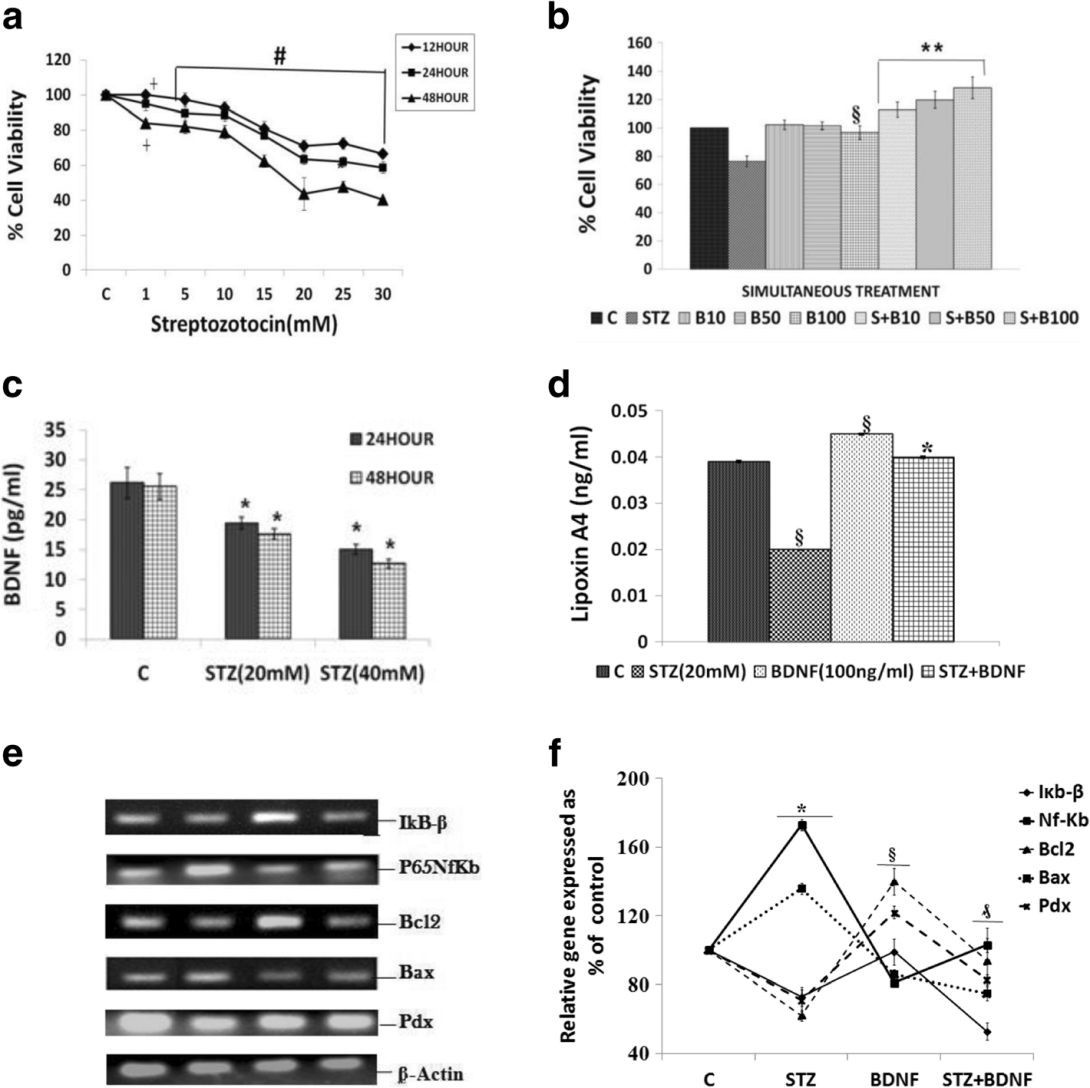
Dose and time optimisation studies with streptozotocin (STZ) on the viability of RIN5F cells in vitro. (**a**) Effect of STZ on viability of RIN-5F cells. ┼*P* ≤ 0.02 and # *P* < 0.001 vs untreated control. (**b**) Effect of simultaneous -treatment with BDNF on STZ (20 mM)-induced toxicity to RIN5F cells. All values expressed as mean ± SEM. ^§^*P* < 0.001 vs untreated control, **P < 0.01 vs STZ-treated group. *P < 0.001 vs respective untreated control (B = brain derived neurotrophic factor). All the above set of experiments were done in triplicate on three separate occasions (*n* = 9) and all values expressed as mean ± SEM (**c**) Measurement of BDNF (pg/ml) by ELISA in the supernatant of RIN5F cells treated with STZ using two different concentrations of STZ (20 and 40 mM) and at the end of two incubation periods (24 and 48 h). All values are expressed as mean ± SEM. *P < 0.001 compared to control. (**d**) Measurement of LXA4 (ng/ml) by ELISA in the supernatant of RIN5F cells treated with STZ (20 mM) and BDNF (100 ng/ml). All values are expressed as mean ± SEM. *P < 0.001 compared to control and STZ treatment. §*P* < 0.001 vs untreated control, *P < 0.001 vs STZ-treated group (**e**) and (**f**) Effect of STZ (20 mM)-induced changes in the mRNA expression of IkB-β; P65NF-κB; Bcl2; Bax; and Pdx in RIN5F cells. Cells were treated simultaneous with BDNF (100 ng/ml) and STZ for 24 h. IkB-β: §*P* < 0.001 vs untreated control P65NFκB: *P < 0.001 vs untreated control Bcl2: *P < 0.001 vs untreated control. Bax: §*P* < 0.05 vs untreated control; Pdx: *P < 0.001 compared to untreated control

Fasting blood glucose was measured by using Accu-Check blood glucose meter on 10th, 20th and 30th day from day 1 of the injection of STZ. The animals were confirmed to have developed diabetes when fasting blood glucose levels were > 250 mg/Dl. Body weight and food consumption was measured twice a week. The total duration of the study was 30 days from the day of the injection of STZ (Fig. 1a). At the end of 30 days, animals were sacrificed to collect blood and various tissues for further studies. All samples were stored at − 80^0^ C till further analysis.

#### Estimation of nitric oxide, lipid peroxides and antioxidant enzymes

Nitric oxide, lipid peroxides and antioxidant enzymes: super oxide dismutase (SOD), catalase, glutathione peroxidase (GPX), glutathione-S-transferase (GST) were estimated in the plasma and lysates of the collected pancreas and brain tissues samples as described previously.

#### Estimation of plasma BDNF, IL-6, LXA4 and TNF-α

The levels of BDNF were measured from the plasma collected at the end of experiment (day 30) using Chemikine Sandwich ELISA kit, Millipore, USA (CYT306). Plasma TNF-α (by using Quantikine TNF-α Immunoassay ELISA kit (RTA00), R&D Systems, MN, USA), LXA4 (EA 45, Oxford biomedical research, USA) and IL-6 were measured by using Abcam (Ab100772) Rat IL-6 ELISA kit, Kendall Square, Cambridge, MA USA, on day 30 of the study employing manufacturer’s protocol.

#### Western blot analysis

Protein lysates were obtained by homogenizing the pancreatic and brain tissues (whole brain tissue) with T-PER (Tissue-protein extraction lysis solution) (50 mg/500 μl lysing solution) along with 10 μl protease inhibitor cocktail and 10 μl phosphatase inhibitor. Homogenization was done under ice and centrifuged at 10,000×g for 5 min to collect the debris. Protein quantification was done by Bradford assay and 40 μg of protein was loaded into the SDS-PAGE. The separated protein samples were transferred to the nitrocellulose membrane by using Bio-Rad Transblot turbo transfer system. After transfer, membrane was blocked by using 5% Bio-Rad blocking reagent. Blocked membrane was incubated with 1:1000 dilution primary rabbit antibodies (Cell Signaling Technology, USA) for 12 h at 4 °C. After treatment, the membrane washed and incubated with secondary antibody (1: 20,000) treatment for an hour. Bands were observed in the chemiluminescence document reader, after addition of Immobilon Western chemiluminescent HRP substrate at standardized exposure time. Bands of the two groups were analyzed by densitometry analysis compared to β-Actin (Major Science image analysis software).

##### Statistical analysis

All studies were repeated thrice each time in triplicate (in vitro) Results are expressed as mean ± SEM and all values obtained were analyzed employing paired t test with equal variance in Microsoft Excel statistical analysis tool.

## Results

### MTT studies

#### Effect of STZ on viability of RIN5F cells

##### Dose and time optimization studies

Studies with STZ showed that exposure of RIN5F cells to 1–30 mM for 12, 24, and 48 h, there was a significant decrease (*P < 0.001*) in their survival in a dose-dependent fashion as shown in Fig. 1a. STZ at 20 mM for 24 h reduced the viability of RIN5F cells to ~ 60%. Based on these results, all subsequent studies were performed using 20 mM and an incubation time of 24 h.

#### Effect of BDNF on STZ-induced cytotoxicity

The results of this study shown in Fig. 1b revealed that though BDNF is effective in preventing the cytotoxic action of STZ at all the three doses (10 ng, 50 ng and 100 ng/ml) tested, the most effective doses are 50 ng (*P* < 0.01) and 100 ng (*P* < 0.001) (100 ng > 50 ng) when BDNF was added to RIN5F cells simultaneously with STZ. Based on these results, all further experiments with STZ were performed using this treatment schedule.

### Effect of BDNF on LXA4 secretion by STZ treated RIN5F cells in vitro

Since BDNF can protect RIN5F cells from the cytotoxic action of STZ, we next studied whether STZ interferes with the synthesis and secretion of BDNF by RIN5F cells in vitro. The results of this study, given in Fig. 1c shows that STZ indeed decreases BDNF secretion by RIN5F cells. In a previous study [34, 35], we observed that LXA4, a potent anti-inflammatory molecule derived from arachidonic acid (AA, 20:4 n-6) protects RIN5F cells from the cytotoxic action of STZ and STZ inhibited protection and secretion of LXA4 by RIN5F cells. Hence, we studied whether BDNF can restore LXA4 secretion inhibited by STZ to normal by RIN5F cells in vitro. Results of this study given in Fig. 1d indicated that under the conditions employed, STZ significantly decreased (*P* < 0.01, *P* < 0.001) the formation and secretion of LXA4 that can be restored to normal by BDNF in RIN5F cells.

### Effect of BDNF on the expression of PDX1, P65 NF-kB and IKB in STZ treated RIN5F cells

The effect of BDNF on mRNA expression of the inflammatory genes (NF-kB and IKB-β) and pancreatic homeobox (Pdx1) regulator and apoptotic genes (Bcl2 and Bax) in RIN5F cells were measured by semi-quantitative PCR. Pdx1 is a homeobox protein expressed in beta pancreatic cells that maintains and expresses the endocrine function of pancreas. The results of these studies given in Fig. 1e and f revealed that STZ significantly increased (*P < 0.05*) Nf-kB mRNA and reduced that of IkB-β and Pdx (*P < 0.05)* expressions in RIN5F cells and these reverted to normal on supplementation with BDNF to RIN5F cells. These results suggest that STZ enhances inflammatory events in RIN5F cells and thus causes damage to β cells.

### STZ-induced changes in antioxidants and their restoration to normal by BDNF

**Fig. 2:**
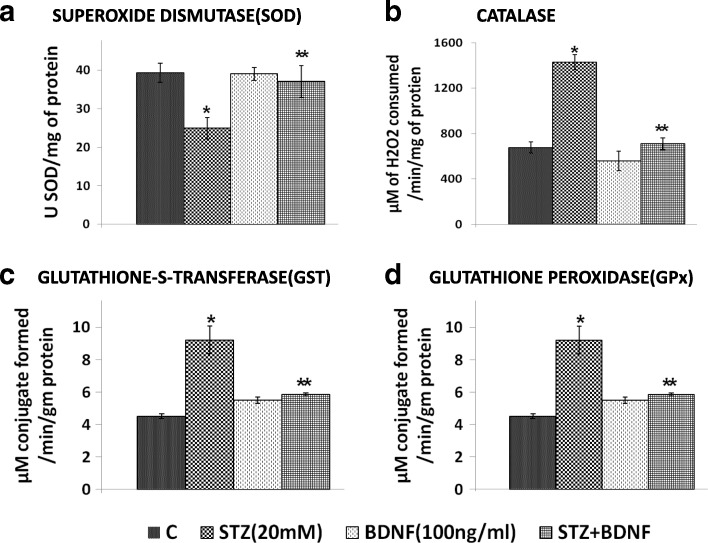
Effect of STZ and BDNF on levels of different anti-oxidants in lysates of RIN5F cells upon simultaneous treatment with BDNF (100 ng/ml) on STZ (20 mM)-induced toxicity to RIN cells. All values expressed as mean ± SEM. § *P* < 0.05, *P < 0.001 vs untreated control, and **P < 0.001 Versus STZ -treated group. **a**. Superoxide disutase; **b**. Catalase; **c**. Glutathione S-Transferase; **d**. Glutathione peroxidase

**Fig. 3:**
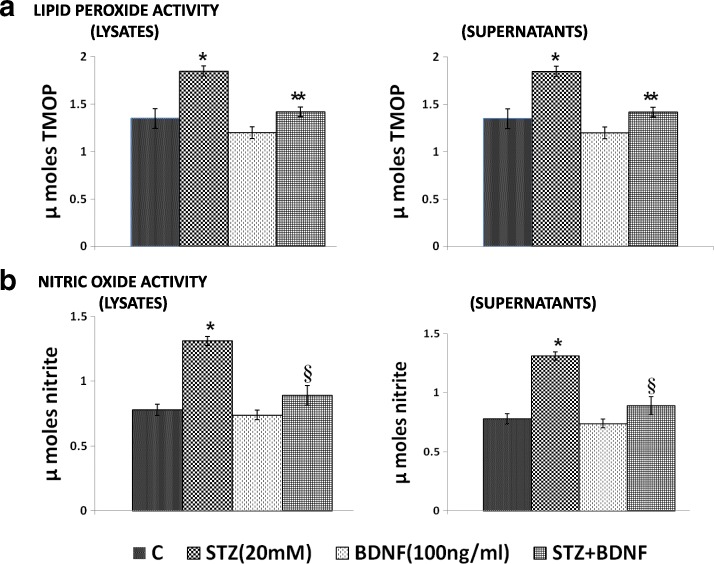
Effect of STZ and BDNF on levels of lipid peroxides (3**a**) and nitrate in lysates and supernatants (3**b**) of RIN cells. All values expressed as mean ± SEM. *P < 0.001 vs untreated control and §*P* < 0.001,*P < 0.001 vs STZ -treated group. AA, arachidonic acid

To know whether cytotoxic action of STZ on RIN5F cells is due to changes in the concentrations of various anti-oxidants, we studied the activity of SOD, catalase, glutathione S-transferase, glutathione peroxidase (Fig. 2) and alterations in lipid peroxides and nitric oxide (Fig. 3) in these cells and pancreas of STZ-induced type 2 diabetes animals (Table 1). STZ-induced significant changes (*P*<0.05) in the concentrations of SOD, catalase, glutathione S-transferase, glutathione peroxidase, lipid peroxides and nitric oxide in RIN5F cells and pancreas of type 2 DM animals reverted to normal by BDNF (100 ng/ml).

**Table 1:** Concentrations of various anti-oxidants, lipid peroxides and nitric oxide in pancreatic tissue in vivo

Summary of the analysis of various anti-oxidant/lipid peroxides and nitric oxide levels in pancreas of stz treated diabetic rats							
Tissue	Group	SOD (Units/mg protein)	CAT (mM H2O2/min/mg protein	GST (mM/min/gm protein)	GPX (mM/min/gm protein)	LPO (μM TMOP)	Nitric oxide (μM nitrite)
Pancreas [in vivo)	Control	36.9 ± 1.9	911 ± 62.8	19.58 ± 0.40	116.5 ± 2.6	1.3 ± 0.05	1.1 ± 0.02
	STZ [65 mg/kg)	74.4 ± 3.2*	1494 ± 41*	54.8 ± 0.36*	191 ± 3.9*	1.5 ± 0.1*	1.6 ± 0.8*

Superoxide dismutase (SOD) is expressed as U SOD/mg of protein; Catalase (CAT) is expressed as μM of H_2_O_2_ consumed/minute/mg of protein; Glutathione-S-transferase (GST) is expressed as μM conjugate formed/ minute/gm of protein; Glutathione peroxidase (GPX) is expressed as μg of glutathione consumed/minute/gm of protein. Lipid peroxides formed are expressed as μmoles of TMOP formed; Nitric oxide formed is expressed *P < 0.05, vs untreated control Vs respective STZ-treated group. All the above set of experiments done on *n* = 8 animal experiment in vivo. All values expressed as mean ± SEM

### Induction of type 2 DM

**Fig. 4:**
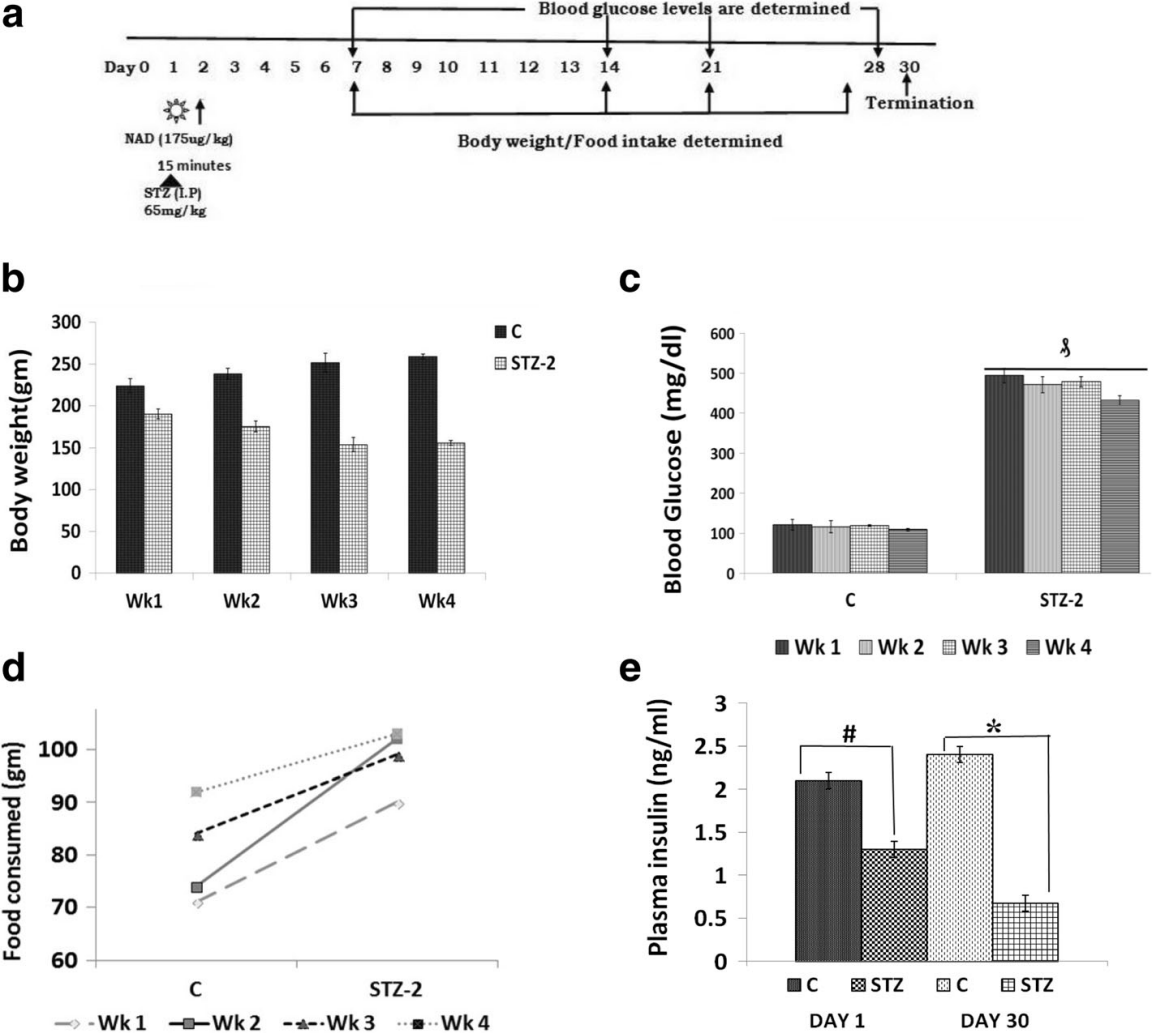
(**a**) STZ-induced type 2 diabetes protocol. Animals were housed for 1 week for acclimatization after which single I.P injections of STZ was given and blood glucose levels were estimated once in 10 days until the end of the study. **(b)** Body weight and (**d**) food consumption of rats treated with STZ as well as control was measured weekly once. (**c**) Blood glucose levels were measured once in 7 days until the end of the study. ^₰^*P ≤ 0.01* compared to control values; (**e**) Plasma insulin levels were measured on Day 1 after STZ (i.p) injection and on Day30, study was terminated, and organs were collected for various molecular and biochemical analysis

To verify whether the in vitro results are relevant to an in vivo situation, we measured plasma BDNF, LXA4 and inflammatory cytokines IL-6 and TNF-α in STZ-induced type 2 DM Wistar rats. The protocol of induction of type 2 DM by STZ in Wistar rats is in Fig. 4.

#### Changes in the blood glucose levels and body weight of STZ-induced type 2 DM rats

STZ injection (65 mg/kg) resulted in the development of type 2 DM as evidenced by significantly increased (> 400 mg/dl, *P* < 0.001 compared to control) blood glucose levels. The fasting blood glucose levels and the body weight of the rats in the two groups were similar prior to the experiment. The increased blood glucose levels of the rats injected with STZ remained in a hyperglycemic state throughout the period of the study. However, no change in the blood glucose levels was detected in the control group (Fig. 4c). The body weight of the rats in both the groups increased over time throughout the experimental period. At the end of the first week after STZ injection, the body weight of the diabetic rats was significantly lower (*P* < 0.001) than that of the control (Fig. 4b). Plasma Insulin levels were significantly (*P* < 0.05) lower in STZ treated group compared to control (Fig. 4e). These results confirmed the validity of the rat model of type 2 diabetes used in the present study.

#### Expression of Akt, mTOR and PI3K in the cerebral cortex of STZ-induced diabetic rats

**Fig. 5:**
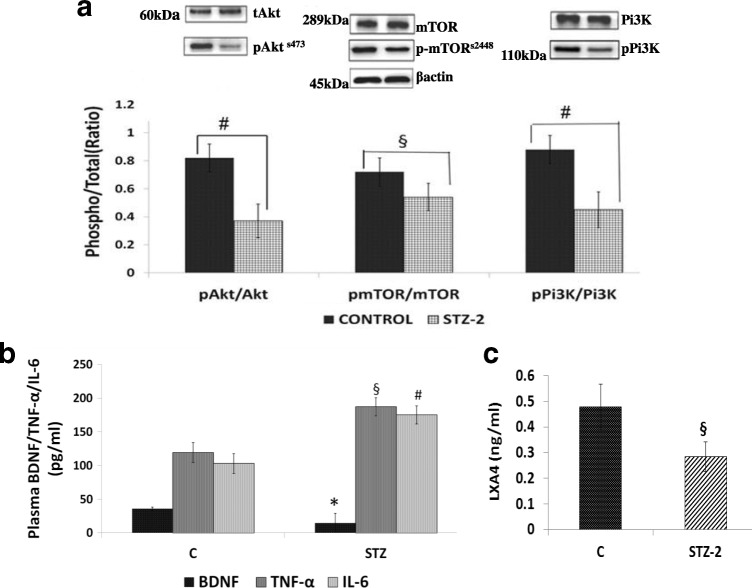
(**a**) Expression of cell survival signaling proteins in the brain of STZ-induced type 2 DM animals. Proteins studies by Western blot include: (pAkt/tAkt), # *P* < 0.001 vs untreated control; (pmTOR/mTOR) §*P ≤ 0.01* vs untreated control. (pPi3K/PI3K) # *P* ≤ 0.01 vs untreated control along with beta Actin. All values are expressed as mean ± SEM. **(b**) Plasma BDNF/TNF-α/IL-6 levels**.** All values are expressed as mean ± SEM. *§P ≤ 0.05* compared to respective control ^₰^*P ≤ 0.01* compared to control. ^*^P ≤ 0.05 compared to control. (**c**) Plasma LXA4 levels in STZ-induced type 2 DM animals compared to control. Data are mean ± SEM. *§P ≤ 0.01* compared to control

Previously, we observed that STZ-induces apoptosis of pancreatic β and neuronal cells (7, 34]. But the exact mechanism by which neuronal apoptosis is induced is not known. Hence, in the present study we evaluated whether in STZ-induced type 2 DM rats there is a role for PI3K pathway in the neuronal apoptosis. Western blot analysis performed revealed that pAkt/Akt, pmTOR/mTOR and pPI3K/PI3K were all significantly decreased (*P* < 0.001) in the cerebral cortex of STZ-induced type 2 DM compared to control (Fig. 5a). This indicates that inhibition of the PI3K pathway has a role in neuronal apoptosis of STZ-induced diabetic rats.

#### Plasma BDNF, LXA4, IL-6 and TNF-α levels

Plasma levels of BDNF were significantly reduced (*P* < 0.01), while IL-6 and TNF-α were significantly elevated in STZ-induced type 2 DM animals (Fig. 5b),while that of LXA4 are reduced (Fig. 5c) compared to control, when measured on day 30th of the study. Since BDNF is a neurotrophic factor that is needed for the survival of neurons and IL-6 and TNF-α are pro-inflammatory and cytotoxic molecules, these results imply that their alterations could lead to an increase in neuronal apoptosis.

#### Expression of iNOS, Foxo-1, Nf-kB, PPAR and Gsk-3β in the brain of STZ-induced type 2 DM rats

**Fig. 6:**
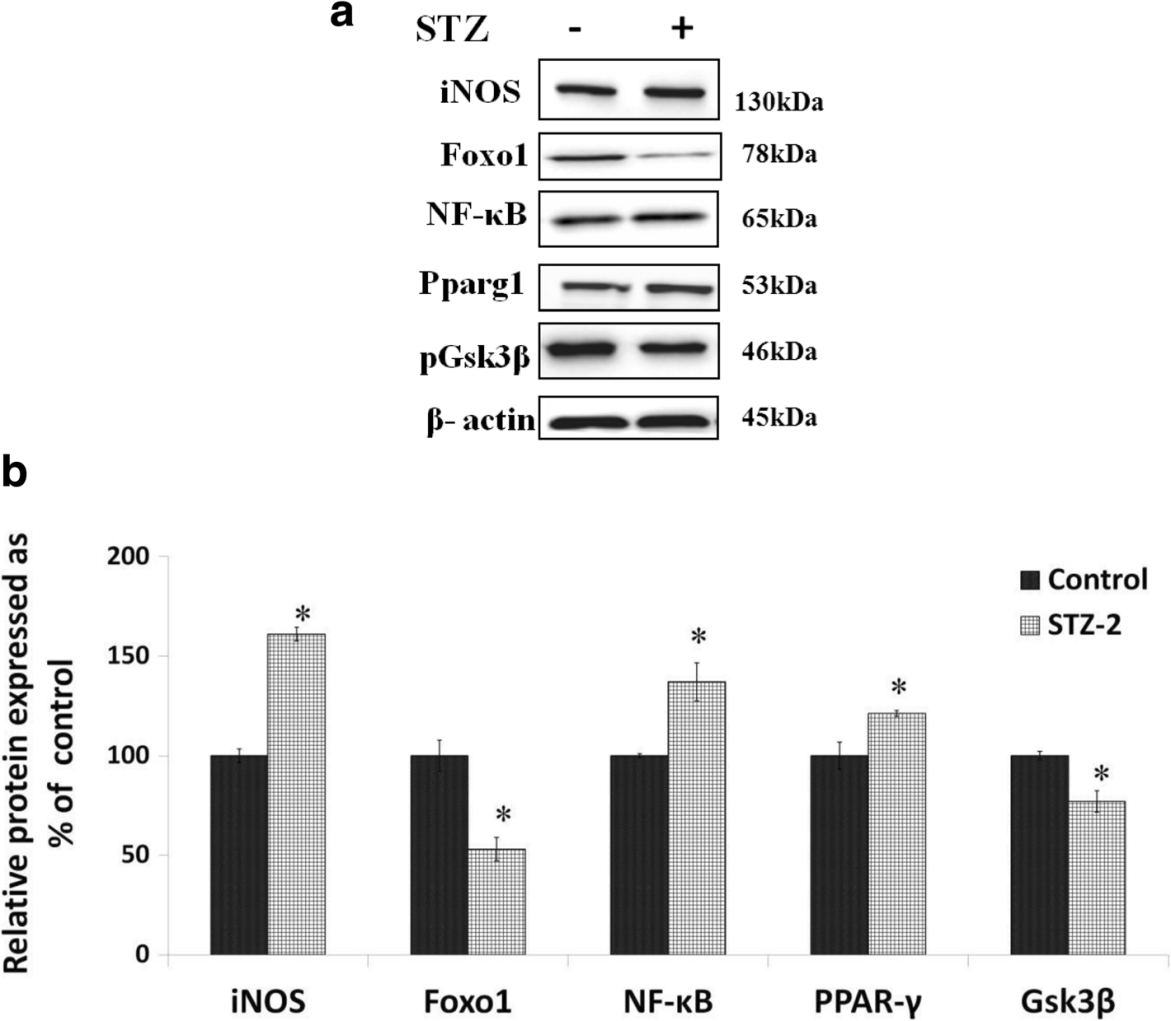
**a** Expression of inflammatory and apoptotic genes in the brain of STZ-induced type 2 DM animals. **b** Expression of iNOS, pFoxo1, Nf-κB, PPAR-g1, pGsk3β proteins *P ≤ 0.05 compared to untreated control. All values are expressed as mean ± SEM

To confirm that STZ-induced type 2 DM is associated with inflammation in the brain, Western blot was performed to document the protein expression of iNOS, Foxo1, Nf-kB, PPAR-γ and Gsk3-β. These results revealed that the expressions of iNOS, NF-kB and PPAR-γ were upregulated while that of Foxo-1 and Gsk3-β downregulated in the DM group compared to control (Fig. 6).

## Discussion

The focus of the present study was to explore mechanisms underlying β-cell apoptosis and neurodegeneration in STZ-induced type 2 DM animals. We hypothesized that impairment of the PI3K/Akt/mTOR pathway may have a role in this process. The results of the present study revealed that STZ treatment significantly reduced body weight, induced hyperglycemia and reduced circulating insulin levels indicating the establishment of type 2 DM. There was a significant increase in the expression of increased (*P < 0.05*) Nf-kB mRNA and reduced that of IkB-β and Pdx (*P < 0.05)* in RIN5F cells and reduction in PI3K-Akt-mTOR signalling in brain samples of STZ-treated animals (Figs. 5a) suggesting that STZ can increase oxidative stress and activate apoptotic genes and decrease the survival of pancreatic β (in vitro) and neuronal cells (in vivo). Thus, STZ may block signal transduction via Akt and/or interfere with hypothalamic signaling either upstream (IRS-2) or down-stream (PKB) of PI3K in diabetic rats. A major downstream target of Akt is mTOR, which has a key role in cell survival, growth, protein synthesis and cellular metabolism. Our findings showed that, after treatment with STZ, there was a  significant reduction in phosphorylated mTOR expression in diabetic brains. We noticed a significant reduction in the expression of pGsk3β and Foxo1 in the brains of STZ treated animals (Fig. 6).

It is evident from these results (Figs. 5 and 6) that STZ increased the expression of iNOS, Nf-kB and PPARγ but reduced that of pGSK-3β in the brain confirming a significant role for inflammatory process in neuronal apoptosis. Furthermore, PI3K has a major role in insulin function, mainly by the activation of the Akt/PKB and the PKC cascades. Activated Akt induces glycogen synthesis through inhibition of GSK-3β protein synthesis via mTOR and its downstream elements and improves cell survival through inhibition of pro-apoptotic agents (Bax). Akt phosphorylates and directly inhibits Foxo1 transcription factors, which also regulate metabolism and autophagy. These findings implicate dysregulation of insulin signaling via the IRS-PI3K pathway is a key determinant of the glycemic response seen in uncontrolled diabetes. We have demonstrated for the first time a reduction in insulin signaling pathway proteins PI3-K/Akt/mTOR pathway in the brain of STZ treated rats that may be implicated as a dominant factor in neurodegeneration leading to cognitive dysfunction seen in diabetics.

One of the criticisms of animal models used for the study of type 2 DM is the fact that diabetes can be cured in rodent models, but these results are not always extrapolatable to humans to develop appropriate therapeutic interventions. In this context, we wish to emphasize that STZ-induced type 2 DM is a well-accepted model to study pathobiology of type 2 DM and following the results obtained in this model appropriate studies are planned in humans. It is interesting to note that the final event involved in the development of type 2 DM is β-cell failure/death, which is also seen in type 1 DM [37]. This is the reason as to why the β-cell toxin, STZ, is used to induce both type 1 and type 2 DM in experimental animals. Since obesity precedes the development/diagnosis of type 2 DM, high fat diet (HFD)/STZ protocol favors mimicking of type 2 DM. In an extension of the present study, we also performed a similar study using HFD/STZ model of type 2 DM. In this animal model of type 2 DM we noted a decreased plasma LXA4 levels, and pro-inflammatory events as seen in the present study [unpublished data]. Furthermore, our recent studies revealed that plasma concentrations of AA and LXA4 are low in those with type 2 DM [38, 39] implying that the results obtained in the present study are extrapolatable to humans. Based on these results, we are now planning to test the efficacy of AA in the prevention of type 2 DM in humans.

## Conclusion

The results of the present study showed that inflammation plays a significant role in the apoptosis of pancreatic β cells and neuronal dysfunction. It is likely that suppression of neuronal PI3K/Akt/mTOR pathway occurs in STZ-induced type 2 DM that may play a role in the pathobiology of hyperglycemia and neurodegeneration and dementia seen in type 2 DM.

## Abbreviations

Bax: BCl2 associated x-protein
Bcl2: B-cell lymphoma
BDNF: Brain-derived neurotrophic factor
CA1: Cornu ammonis
DG: Dentate gyrus
EDTA: Ethylene diamine tetra acetic acid
Foxo 1: Fork head box 01
GPX: Glutathione peroxidase
Gsk-3β: Glycogen kinase synthase Beta
GST: Glutathione-S-transferase.
HFD: High fat diet
IkB-β: Nuclear factor of kappa light polypeptide gene enhancer B-cells
IL-6: Interleukin-6
iNOS: Inducible nitric oxide synthase
mTOR: Mechanistic target of rapamycin
NaCl: Sodium chloride
NAD: Nicotinamide
Nf-kb: Nuclear factor kappa b
Nrf2: Nuclear factor erythroid2-related factor2
pAkt: Phosphorylated Akt
Pdx1: Pancreatic and duodenal homeobox-1
PI3-K: Phosphatidylinositol 3-kinase
PVN: Paraventricular nucleus
RIN5F: Rat insulinoma
SOD: Superoxide dismutase
STZ: Streptozotocin
tAkt (Total Akt): Serine/Threonine-specific protein kinase
TNF-α: Tumour necrosis factor alpha
TPER: Tissue protein extraction regent
